# Professionals Evaluating Clients’ Suitability for Digital Health and Social Care: Scoping Review of Assessment Instruments

**DOI:** 10.2196/51450

**Published:** 2023-11-30

**Authors:** Anu-Marja Kaihlanen, Lotta Virtanen, Emma Kainiemi, Tarja Heponiemi

**Affiliations:** 1 Finnish Institute for Health and Welfare Helsinki Finland

**Keywords:** access, accessibility, assessment, clients, digital health, digital social care, eHealth, evaluation tool, evaluation, evaluator, instrument, knowledge synthesis, patients, professionals, review methodology, review methods, scoping, social care, suitability

## Abstract

**Background:**

Increased digital health and social care services are generally considered to improve people’s access to services. However, not everyone can equally access and use these resources. Health and social care professionals should assess clients’ suitability for digital solutions, but to succeed, they need information about what to evaluate and how.

**Objective:**

This scoping review aimed to identify evaluation tools that professionals can use when assessing clients’ suitability for digital health and social care. We summarized the dimensions and the practical usefulness of the instruments.

**Methods:**

The MEDLINE (Ovid), CINAHL, Web of Science, and ASSIA databases were searched in February 2023 following the Joanna Briggs Institute’s Manual for Evidence Synthesis. Studies were included if they focused on health and social care clients and professionals, examined clients’ suitability for using digital health or social care, and applied related assessment methods in the direct client work of professionals. Studies focusing primarily on instruments intended for research use without clear applicability to professionals’ practical contexts were excluded. Details of the eligible studies were extracted, and qualitative content analysis according to the research objectives was performed.

**Results:**

A total of 19 articles introducing 12 different assessment instruments intended for the health care context were included in the review. No instruments were found for evaluating the suitability for digital social care. The instruments contained 60 dimensions of the client’s suitability for digital health, which reflected four perspectives: (1) skill-based suitability, (2) suitability based on general ability to maintain health, (3) suitability based on attitude and experience, and (4) suitability based on practical matters. The described practical usefulness of the instruments included professionals’ possibility to (1) identify clients most in need of education and support, (2) direct and recommend the right clients for the right digital services, (3) ensure that clients can use digital health, (4) improve effectiveness and maximize the provision of digital health, (5) develop and redesign services, and (6) empower clients.

**Conclusions:**

Based on the diverse assessment instruments available and the dimensions they measure, there seems to be no comprehensive evaluation tool for assessing clients’ prerequisites to use digital solutions. It is important to further develop comprehensive screening tools applicable to professionals’ busy work (both in health and social care) with defined threshold values for suitability.

## Introduction

To achieve the vision of better health and well-being for all, the need to strengthen health and social care services by applying digital technologies is well-recognized worldwide [1,2]. One of the primary goals of digitalization in health and social care is increased access to services [3,4]. Digital health and social care refers to the use of digital technologies to support the delivery of services with the aim of promoting individuals’ health and well-being and managing health conditions or risks [1,5]. Digital health and social care encompass a wide range of technologies, such as remote receptions and interventions, mobile health, websites supporting health and well-being, client and patient portals, remote monitoring systems, and wearables. These technologies have aimed to increase flexibility in the provision of services, help clients access services remotely, and reduce the need for in-person visits [6]. Moreover, digital technologies can enable clients to communicate effectively and easily with their service providers [7] and manage their own health and well-being more actively [4,8], which also belong to the strategic goals of digitalization [1,9].

The results achieved from the provision of digital technologies do not only depend on their quality but also on clients and health or social care professionals, who have a significant role as end users [10,11]. While increased digitalization is generally seen as increasing access to services, not everyone can use these resources equitably. Numerous factors, such as insufficient skills, available equipment and space, lack of interest or knowledge of available digital services, and lack of support, among others, can make it difficult for many to benefit from digital health and social care [12,13]. In the health care context, having sufficient digital health literacy (also known as eHealth literacy [eHL]) has been seen as an essential prerequisite [14]. It is defined as a set of skills required in the use of digital technologies to search, acquire, understand, evaluate, communicate, apply, and create health-related information to improve the quality of life [15].

Professionals have a key role in promoting the use of digital health and social care, for example, by referring clients to digital services [16] and actively providing information, support, and encouragement for use [17]. Increased digitalization has also induced other new tasks for professionals, such as identifying whether clients’ situations and circumstances favor the use of digital services [18]. When considering the use of a digital solution, professionals need to be able to evaluate the client’s individual needs and potential barriers [19], as well as their willingness and capabilities for use [20]. To successfully fulfill this task, professionals should know what they should evaluate and how. Otherwise, professionals’ own competence, attitudes, or preferences can determine whether they recommend digital services to their clients and promote their use [11,21].

A considerable number of studies have been conducted to identify factors that could predict clients’ adoption and use of digital services, such as telemedicine consultations [22], mobile health tools [23], and patient portals [16]. The information obtained on the associated factors could, in principle, be used by professionals when evaluating the client’s suitability for using digital health and social care. However, it may also lead to making generalizations and assumptions about suitability without individual assessment. Stereotypical thinking about suitable users (eg, based on age) increases the risk that some clients who could use and benefit from digital services might remain unrecognized. Therefore, appropriate evaluation tools are needed for the various factors that may affect the client’s use of digital services.

The aim of this review was to identify available evaluation tools that health and social care professionals can use when assessing a client’s suitability for digital health and social care. The review addresses the following research questions:

1. What instruments are available for health and social care professionals to assess a client’s suitability for digital health and social care?
2. What dimensions (factors) are included in the instruments to assess a client’s suitability for digital health and social care?
3. What is the described practical usefulness of the instruments for the professionals’ work?

## Methods

We conducted a scoping literature review by following the Joanna Briggs Institute’s Manual for Evidence Synthesis [24].

### Eligibility Criteria

Eligibility criteria were defined based on the population, concept, and context framework [24]. Population included were health and social care clients and professionals (ie, care providers). Concept was clients’ suitability for using digital health or social care and methods that can be used to assess it, such as existing instruments or guidelines. Context was the direct client work of health and social care professionals for which the assessment method had to be intended or applied. Studies were excluded if the assessment method was intended mainly for research use (such as national or cross-national population–based surveys) and if its usefulness or applicability to professionals’ work or a practical context was not described.

### Types of Sources

This review considered peer-reviewed empirical studies (quantitative, qualitative, or mixed methods), systematic literature reviews, and papers presenting instrument development or evaluation. Other types of papers, such as policy documents, protocols, or discussion papers, were excluded as they were not seen to provide as reliable information as peer-reviewed scientific publications. Due to the rapid progress of digitalization in the past decade [25], we limited the search to studies published between 2012 and 2023 with an English abstract and full text.

### Search Strategy

A total of 4 databases were selected, which were assumed to contain relevant articles to answer the research question: MEDLINE (Ovid), CINAHL, Web of Science, and ASSIA. First, a preliminary search was conducted in CINAHL to identify relevant search terms. Then, the expertise of a research librarian was used in the optimization of search words and strategies in the selected databases. Search strategies were tested in CINAHL and MEDLINE (Ovid) and refined before performing final searches in all 4 databases in February 2023. The reference lists of the included studies were screened for potential additional publications. The search strategy for databases is presented in Table S1 in Multimedia Appendix 1 [26-44].

### Study Selection

The search records were transferred to the reference management system Zotero (Corporation for Digital Scholarship) to remove duplicates. First, 2 reviewers (AMK and LV) independently conducted title and abstract screenings of the publications. Then, both reviewers screened the full texts of the publications and provided reasons for their exclusion. In both phases, studies with conflicting decisions were rescreened and solved by consensus between the reviewers.

### Data Extraction

The following data were extracted from the studies: (1) authors, publication year, and country; (2) study aim; (3) methodology and participants; (4) instruments, dimensions, and number of items; (5) associations of the values obtained with the instrument with the use of digital health and social care or other relevant findings; and (6) practical usefulness for professionals.

### Data Analysis

As recommended in scoping reviews, in addition to counting frequencies for the studies’ demographics, we used qualitative content analysis for each research question [24,45]. The method allowed for identifying and creating an overall picture of the key content areas of the assessment of the client’s suitability for digital health and social care, as well as the practical usefulness of the instruments for professionals’ work. First, all the individual expressions corresponding to the research questions were extracted from the text, and their content was simplified and clarified. Then, these “codes” were classified into categories based on similarity [46]. Preliminary categorization was done by the first author (AMK), after which the names and contents of the categories were evaluated by the coauthors to ensure consensus.

### Retrieval of Studies

**Figure 1:**
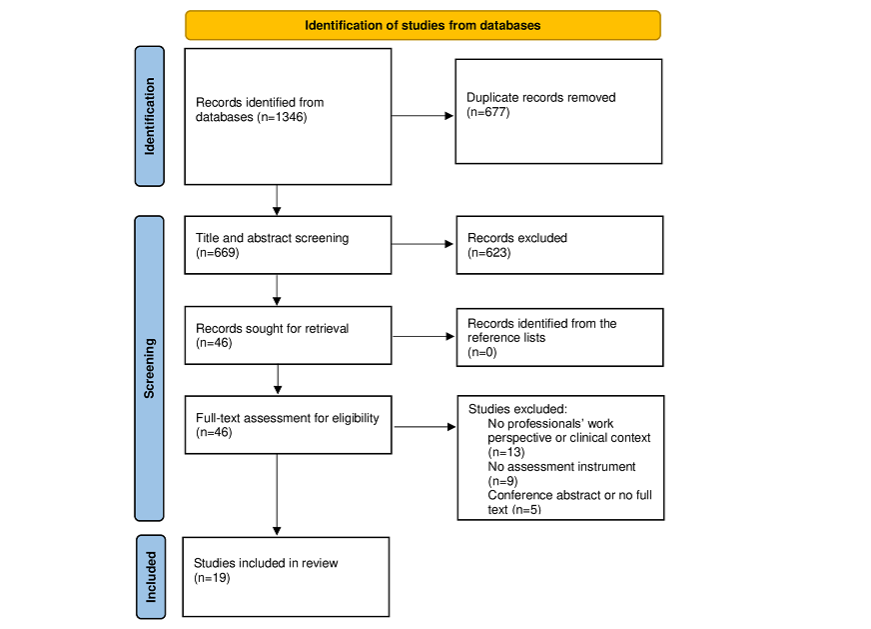
PRISMA (Preferred Reporting Items for Systematic Reviews and Meta-Analyses) flow diagram describing the selection process.

A total of 1346 studies were identified (MEDLINE [Ovid], n=418; CINAHL, n=324; Web of Science, n=513; and ASSIA, n=91). After removing duplicates (n=677), a total of 669 studies were included in the title and abstract screening. Based on the inclusion and exclusion criteria, 623 studies were excluded, and 46 were retrieved for full-text evaluation. Finally, 19 studies were included in the data extraction process. Figure 1 illustrates the flowchart of the selection process.

## Results

### Description of the Studies

Of the studies included (n=19), a total of 6 originated from North America, and other regions were Asia (n=5), Europe (n=4), Australia (n=3), and the Middle East (n=1; Table S2 in Multimedia Appendix 1). The studies were published between 2012 and 2023, with nearly half of them (n=9) within the past 3 years (2020 or after). Most of the studies (n=15) aimed to adapt a certain measuring instrument to a specific client group and conduct validity and reliability testing (ie, psychometric properties and psychometric validation). The rest aimed to develop a measuring instrument (n=2), evaluate the usability of combining several instruments (n=1), and evaluate the content and response process of an instrument (n=1). All studies were conducted in the health care context, and none of the studies dealt with the client’s suitability for digital social care. Thus, all studies considered instruments assessing clients’ suitability for using digital health. Most of the studies used quantitative methods (n=17), only 1 used a qualitative method, and 1 used mixed methods. More than half of the included studies were targeted at clients with different health conditions or disabilities, such as chronic diseases (n=6), cardiovascular diseases (n=3), cancer (n=1), and autism (n=1). A total of 5 studies did not specify the condition of the participants but included clients visiting hospital outpatient clinics, primary care clinics, family practice or internal medicine physicians, or clients undergoing day surgery or needing magnetic resonance imaging or computerized tomography. Additionally, in 3 studies, participants were identified from registers.

### Instruments Assessing Client’s Suitability for Using Digital Health

The studies introduced 12 instruments that were described to measure the client’s potential and suitability for using digital health from slightly different perspectives (Table 1). The most-used instrument in the studies was the eHealth Literacy Scale (eHEALS; n=8). A total of 3 studies used the eHealth Literacy Questionnaire, and 2 studies included the Health Literacy Questionnaire. All the remaining studies included a different instrument: the eHealth Literacy Scale, eHealth Literacy Assessment Toolkit (eHLA), Readiness and Enablement Index for Health Technology Instrument (READHY), Condition-Specific eHealth Literacy Scale for Diabetes (CeHLS-D), Digital Health Care Literacy Scale (DHLS), Telehealth Music Therapy Screening Tool, Digital Health Technology Literacy Assessment Questionnaire (DHTL-AQ), Digital Health Literacy Instrument (DHLI), and the Transactional eHealth Literacy Instrument (TeHLI). All the instruments were based on self-assessment, except for DHLI, which also included performance-based items. Table 1 summarizes the description of the instruments, including the context for using them.

**Table 1:** Summary of the instruments included in the studies.

Instrument	General description^a^	Participants and context of the studies using the instrument	Original source or developer of the instrument
eHealth Literacy Scale (eHEALS)	Measures perceived skills and comfort in using digital technology to access, evaluate, and apply eHealth information to address health concerns	Patients with one or more chronic diseases [26,27], patients undergoing day surgery [28], medical imaging outpatients [29], patients diagnosed with heart failure [30], patients receiving internal medicine care [31], patients with considerable risk for cardiovascular disease [32], and older adults participating in a bone health intervention [33]	Norman and Skinner, 2006 [47]
eHealth Literacy Questionnaire (eHLQ)	Measures health literacy of individuals in the context of digital health technologies	Patients with chronic disease [34], clients from primary care medical clinics [35], and patients with cancer [48]	Kayser et al, 2018 [48]
Health Literacy Questionnaire (HLQ)	Measures an individual’s capacity to effectively use health information and services	Patients with considerable risk for cardiovascular disease [32] and patients with cancer [36]	Osborne et al, 2013 [49]
Electronic Health Literacy Scale (e-HLS) and e-HLS-CHI^b^	Multidimensional instrument that measures aspects of interactive literacy and critical evaluation skills in addition to the skills needed to search for and understand the information provided by electronic sources	Patients with stroke conditions [37]	Seckin et al, 2019 [50]
eHealth Literacy Assessment Toolkit (eHLA)	Combines 7 instruments to assess the individual’s competencies related to health and competencies related to digital solutions	Patients from the hospital outpatient clinic and a sample from the general population	Karnoe et al, 2018 [38]
Readiness and Enablement Index for Health Technology (READHY)	Multidimensional tool that characterizes individuals’ level of health technology readiness by combining 3 instruments: eHealth Literacy Questionnaire, selected dimensions from the Health Education Impact Questionnaire, and the Health Literacy Questionnaire	Cancer rehabilitation context	Kayser et al, 2019 [36]
Condition-Specific eHealth Literacy Scale for Diabetes (CeHLS-D)	Measures eHealth literacy specific for people with type 2 diabetes, including cognitive actions for internet diabetes information and abilities to digital communication	People with type 2 diabetes	Lee et al, 2022 [39]
Digital Health Care Literacy Scale (DHLS)	Short 3-item screening tool that measures the basic skills necessary for using digital health services, including telehealth	Caregivers of young children visiting a pediatric primary care clinic	Nelson et al, 2022 [40]
Transactional eHealth Literacy Instrument (TeHLI)	Multidimensional instrument that measures individuals’ perceived skills related to their capacity to understand, exchange, evaluate, and apply health information from various online sources and multimedia	Patients with COPD^c^	Paige et al, 2019 [41]
Telehealth music therapy screening tool (TMTST)	Screening tool that measures clients’ circumstances that can make them more (or less) suitable for telehealth music therapy. The tool also measures factors related to the professionals and their ability to provide telehealth.	Individuals with autism	Williams et al, 2023 [42]
Digital Health Technology Literacy Assessment Questionnaire (DHTL-AQ)	Measures the person’s knowledge- and skill-based competencies required for the use and adoption of digital health technology, services, and data. The focus and context of DHTL-AQ are in technology, including mobile devices and apps, health IT, and telehealth.	Patients with and those without chronic disease	Yoon et al, 2022 [43]
Digital Health Literacy Instrument (DHLI)	Measures a broad range of skills essential to using eHealth applications, including the ability to interact on the internet.	General (Dutch) population	Van der Vaart and Drossaert, 2017 [44]

^a^Detailed information on the dimensions and factors of the instruments and the number of items is presented in Table S2 in Multimedia Appendix 1.

^b^e-HLS-CHI: Chinese version of Electronic Health Literacy Scale.

^c^COPD: chronic obstructive pulmonary disease.

### Dimensions Included in the Instruments to Assess Client’s Suitability

The number of dimensions (factors) per instrument varied between 1 (eHEALS and DHLS) and 13 (READHY), and the total number of items per instrument varied between 3 (DHLS) and 79 (eHLA). Altogether, 60 individual dimensions were included in the instruments (Table 2). Based on the content analysis, the dimensions were formed into 13 subcategories and further into the following four upper categories embodying different perspectives on the client’s suitability for digital health: (1) skill-based suitability, (2) suitability based on general ability to maintain health, (3) suitability based on attitude and experience, and (4) suitability based on practical matters and implementation. Table 2 shows the categorization of the dimensions in the instruments.

The assessment of “skill-based suitability” included the most dimensions (n=24) and consisted of a client’s ability to acquire and process web-based information for health, the technical skills required to use digital services, and the ability to communicate on the internet. A high number of dimensions (n=20) also focused on the assessment of the clients’ “suitability based on general ability to maintain health,” including health literacy (ie, the ability to acquire and process health-related information), health-maintaining activity (ie, the ability to take care of one’s own health and attitude toward illness), knowledge of the health care system, and the social context and network. The third upper category, “suitability based on attitude and experience,” consisted of 11 dimensions that covered clients’ comfort and confidence to use digital health, in addition to safety and trust and attitudes toward digital health. The least dimensions (n=5) were included in the fourth upper category, “suitability based on practical matters and implementation,” which covered the assessment of technological tools and environment for the use of digital health and possible difficulties in using in-person services.

**Table 2:** Categorization of the dimensions in the instruments measuring client’s suitability for digital health.

Upper category and subcategory		Individual dimension (name of the instrument)
**Skill-based suitability**		
	Acquiring and processing web-based information for health	Reaching information sources: Acquiring information from the internet (C-eHEALS^a^) Navigation skills on the web (DHLI^b^) Awareness and knowledge of information and resources (eHEALS^c^) Functional eHL^d^, for example, basic skills in reading and typing about health effectively on the internet (TeHLI^e^) Information-searching skills (DHLI) Evaluating information: Evaluating the reliability of internet-based information (DHLI) Determining the relevance of internet-based information (DHLI) Evaluating information and resources (eHEALS) Digital critical literacy, for example, the ability to evaluate the reliability and relevance of digital health and information (DHTL-AQ^f^) Communication, for example, discussing the information with a health provider, asking where to find credible information (e-HLS^g^) Action, for example, checking the authors or sponsors of the website; Is the topic comprehensively covered? Is the information current and updated (e-HLS)? Processing information: Using technology to process health information (READHY^h^ and eHLQ^i^) Information engagement (eHEALS) Applying information: Cognitive actions for the internet (diabetes) information (CeHLS-D^j^) Translational eHL, for example, the ability to apply health knowledge gained from the internet across diverse ecological contexts (TeHLI)
	Technical skills required to use digital health	Familiarity with technology (eHLA^k^) Ability to use applications and programs with an electronic device independently (DHLS^l^) Ability to set up a video chat with an electronic device independently (DHLS) Ability to solve basic technical issues independently (DHLS) Familiarity with computer usage (TMTST^m^) Digital functional literacy, for example, ability to use an app, knowing app icons, and ICT^n^-related terms (DHTL-AQ) Operational skills to use the computer and internet browser (DHLI)
	Ability to communicate on the internet	Abilities of digital communication (CeHLS-D) Communicative eHL, for example, the ability to collaborate, adapt, and control communication about health with users in social online environments with multimedia (TeHLI) Adding self-generated content to web-based applications (DHLI)
**Suitability based on general health maintenance ability**		
	Health literacy (ie, the ability to acquire and process health-related information)	Functional health literacy, for example, ability to understand health information (eHLA) Health literacy related to health (disease) care (eHLA) Health literacy related to disease prevention (eHLA) Health literacy related to health promotion (eHLA) Having sufficient information to manage one’s own health (HLQ^o^) Appraisal of health information (HLQ) Ability to find good health information (HLQ) Understanding health information well enough to know what to do (HLQ) Ability to actively engage with health care providers (HLQ)
	Health-maintaining activity (ie, the ability to take care of one’s own health and attitude toward illness)	Self-monitoring and insight, for example, the ability to monitor one’s own condition and make appropriate actions to self-management based on responses (READHY and heiQ^p^) Actively managing one’s own health (HLQ) Constructive attitudes and approaches, for example, How does the individual perceive the illness as affecting their life (READHY and heiQ)? Skill and technique acquisition, for example, skills and techniques that help an individual manage disease-related symptoms and health problems (READHY and heiQ) Emotional distress, for example, overall negative affective responses to illness, such as anxiety, anger, and depression (READHY and heiQ)
	Knowledge of the health care system	Understanding of health concepts and language (READHY and eHLQ) Familiarity with health and health care system and terminology (eHLA) Knowledge of health and health care (eHLA) Navigating the health care system (HLQ)
	Individual’s social context and network	Feeling understood and supported by health care providers (READHY and HLQ) Social support for health (READHY and HLQ)
**Attitude and experience-based suitability**		
	Comfort and confidence to use digital health	Ability to actively engage with digital services, for example, comfortable use of digital services for handling information (READHY and eHLQ) Technology confidence (eHLA)
	Safety and trust in digital health	Feel safe and in control, for example, feeling that stored personal data are secured and can only be accessed by authorized persons (READHY and eHLQ) Trust, for example, trusting that internet information is accurate, credible, and better than what most health providers provide (e-HLS) Access to digital services that work, for example, having trust that they work as expected when needed (READHY and eHLQ) Critical eHL, for example, the ability to evaluate the credibility, relevance, and risks of sharing and receiving health information on the internet (TeHLI) Protecting and respecting privacy while using the internet, for example, sharing own private information intentionally or unintentionally (DHLI) Digital services that suit individual needs, for example, perception of whether services are accessible and adapt to the user (READHY and eHLQ)
	Attitudes toward digital health	Motivation to engage with digital services, for example, perception of usefulness (READHY and eHLQ) Incentives for engaging with technology (motivation; eHLA)
**Suitability based on practical matters and implementation**		
	Technological tools and environment for the use of digital health	Access to the internet and computer with a camera and microphone (TMTST) Having a distraction-free environment (TMTST) Having an appropriate space (TMTST)
	Difficulties in using in-person services	Tendency to have social anxiety with others in-person (TMTST) Limited access to an in-person service (TMTST)

^a^C-eHEALS: Chinese version of the eHealth Literacy Scale.

^b^DHLI: Digital Health Literacy Instrument.

^c^eHEALS: eHealth Literacy Scale.

^d^eHL: eHealth literacy.

^e^TeHLI: Transactional eHealth Literacy Instrument.

^f^DHTL-AQ: Digital Health Technology Literacy Assessment Questionnaire.

^g^e-HLS: Electronic Health Literacy Scale.

^h^READHY: Readiness and Enablement Index for Health Technology.

^i^eHLQ: eHealth Literacy Questionnaire.

^j^CeHLS-D: Condition-Specific eHealth Literacy Scale for Diabetes.

^k^eHLA: eHealth Literacy Assessment Toolkit.

^l^DHLS: Digital Health Care Literacy Scale.

^m^TMTST: Telehealth Music Therapy Screening Tool.

^n^ICT: Information and communication technology.

^o^HLQ: Health Literacy Questionnaire.

^p^heiQ: Health Education Impact Questionnaire.

### Practical Usefulness of the Instruments

From the point of view of the practical usefulness of the instruments for professionals’ work, many of the studies emphasized the instrument’s screening features [27,28,33,36,39,40,43], such as being convenient and brief (eHEALS, CeHLS-D, and DHLS), not burdensome for clients, and easy for professionals to use and complete in a busy clinical setting (CeHLS-D and DHTL-AQ). A total of 19 aspects regarding the practical usefulness of the instruments were described in the studies. Based on the content analysis, six categories were formed that represented different perspectives on how the instruments and the information obtained with them could be used in practice: (1) identifying clients most in need of education, assistance, and support; (2) directing and recommending the right clients for the right digital services; (3) ensuring that the patient can use digital health; (4) improving effectiveness and maximizing the provision of digital health; (5) developing and redesigning systems and services; and (6) empowering clients. Table 3 provides a summary of the described usefulness aspects, and more detailed information is provided in the data extraction table (Table S2 in Multimedia Appendix 1).

Only 1 study, by He et al [37], provided a cutoff point for the values obtained with the instrument (ie, what values are considered low or high, or how the values obtained should be interpreted and used in practice), which could help professionals make decisions about the client’s suitability. A total of 7 studies [26,31,33,40,41,43,44] examined whether the results obtained with the instrument are actually associated with the clients’ use of, or ability to use, digital health (ie, criterion validity). Patients with a higher eHL level were reported as having a higher interest in using internet-based information channels [26], a higher likelihood of adopting and using a personal health record [31] and the internet [33], and perceiving sufficient self-efficacy and knowledge to find and use internet-based health information [27] compared to patients with a lower level of eHL. Higher transactional eHL scores were associated with higher active and interactive internet-based health information–seeking, fewer challenges in information-seeking, and higher perceived usefulness of the internet for health-related purposes [41]. A lower digital health literacy score was associated with less experience with digital health and a lower likelihood of owning digital tools [40]. A few studies highlighted the need to investigate these associations and the predictive value of the instruments in the future [27,32,36,38].

**Table 3:** Practical usefulness of the instruments described in the studies.

Perspectives of practical usefulness and the description of usefulness aspects		Instrument
**Identifying clients most in need of educational interventions, assistance, and support**		
	Identifying clients with a low eHL^a^ level [29,39]	CeHLS-D^b^ and eHEALS^c^
	Identifying clients who may be unaware of their need for assistance [44]	DHLI^d^
	Identifying clients who are at risk of being marginalized [36]	READHY^e^
	Helping in targeting interventions and additional support for those most in need [40]	DHLS^f^
	Helping in giving appropriate advice on how to evaluate web-based sources [29]	eHEALS
	Informing about the need to provide recommendations about reliable sources to avoid getting misleading information [39]	CeHLS-D
	Informing about the need to guide clients to reliable web-based sources to reduce their need to evaluate the contents [29]	eHEALS
**Directing and recommending the right clients for the right digital services**		
	Supporting decisions about which clients are eligible to participate and could benefit from particular eHealth interventions or solutions [30,38,42,44]	DHLI, eHLA^g^, TMTST^h^, and eHEALS
	Informing about which clients are likely to use digital health in the future [31]	eHEALS
	Helping in directing clients to services that match their health attitudes, preferences, and skills [41]	TeHLI^i^
	Helping in directing clients to services that will promote their self-care and well-being [27]	eHEALS
**Ensuring that a patient can use digital health**		
	Helping in ensuring clients’ readiness and ability to use and engage with technology [34,36]	eHLQ^j^ and READHY
	Informing about how clients identify, judge, and use digital health resources [37]	e-HLS^k^
	Informing about whether the patient’s eHL level enables the use of digital health in self-care after a procedure [28]	eHEALS
**Improving the effectiveness and maximizing the provision of digital health**		
	Helping in making telehealth therapy more effective (for a specific patient group) [42]	TMTST
	Maximizing professionals’ ability to provide therapy through telehealth [42]	TMTST
	Optimizing the benefits of eHealth with suitable interventions that facilitate the clients’ access, understanding, and use of information [33]	eHEALS
**Developing and redesigning systems and services**		
	Helping to improve the quality and effectiveness of care by designing more adaptive care and health-promoting programs and digital health interventions that better match users’ health needs [34,35,37]	eHLQ and e-HLS
**Empowering clients**		
	Finding ways to better empower clients to take care of their own health with digital resources [26]	eHEALS

^a^eHL: eHealth literacy.

^b^CeHLS-D: Condition-Specific eHealth Literacy Scale for Diabetes.

^c^eHEALS: eHealth Literacy Scale.

^d^DHLI: Digital Health Literacy Instrument.

^e^READHY: Readiness and Enablement Index for Health Technology.

^f^DHLS: Digital Health Care Literacy Scale.

^g^eHLA: eHealth Literacy Assessment Toolkit.

^h^TMTST: Telehealth Music Therapy Screening Tool.

^i^TeHLI: Transactional eHealth Literacy Instrument.

^j^eHLQ: eHealth Literacy Questionnaire.

^k^e-HLS: Electronic Health Literacy Scale.

## Discussion

### Principal Findings

This scoping review aimed to identify available evaluation tools that health and social care professionals can use when assessing clients’ suitability for digital health and social care. More precisely, the purpose was to review the dimensions (factors) included in the instruments and the described practical usefulness of the instruments for professionals’ work. The 19 studies included in the review covered 5 different continents, which reflects the currency and importance of the topic worldwide. A substantial part of the studies was published after 2020, which can be thought to reflect the COVID-19 era. The variety of digital services in health and social care grew exponentially during the pandemic [51,52], simultaneously increasing the need to evaluate the possibilities and challenges of different user groups to use and benefit from these services [12]. This review identified 12 different assessment instruments that could be used for this purpose. Interestingly, none of them were developed or placed in the context of social care. However, this does not mean that the discovered instruments and their dimensions, particularly in areas such as technical skills, remote communication, attitudes, or existing facilities, for example, cannot also be used to assess suitability for digital social care. Since developing and testing entirely new instruments is a lengthy process, it would be beneficial to consider and explore the applicability of the identified suitability dimensions in the context of social work. All the instruments were based on subjective self-assessment, except for DHLI, which additionally included performance-based items that tested the client’s ability to apply the skills in a fictional situation [44]. It is clear that future studies would benefit from considering more objective methods to evaluate the determinants that may influence an individual’s engagement with digital health [14].

From the identified instruments, it was possible to distinguish numerous dimensions that measured different aspects of a client’s suitability for digital health. The highest number of individual dimensions focused on clients’ skills, particularly the ability to access, evaluate, process, and use web-based health-related information, or in other words, digital health literacy, or eHL. The fact that dimensions related to eHL were included in several instruments most likely reflects its perceived weight and importance when assessing a client’s potential to use and benefit from digital health. Several studies reported that the eHL level is associated with the client’s digital service use [26,27,31,33], and a recent literature review also concluded that eHL has a positive correlation with health-promoting behavior patterns [53]. Both of these findings reinforce the relevance of its assessment. However, focusing only on eHL does not provide an overall picture of the skills needed to use digital health. According to this review, professionals should also consider the client’s technical skills (eg, familiarity with technology and computer use and the ability to use apps) and the skills needed for internet-based communication, which is seen as an essential part of transactional eHL [41,54].

In this review, the client’s general ability to maintain health emerged as another central assessment area. This finding is logical because, as digital health technologies become more common, clients are increasingly expected to actively participate in managing their health and well-being by using them [1,4]. Thus, clients need sufficient health literacy in the context of digital health [55]. This refers to multiple competencies related to “accessing, understanding, appraising, and applying health information in the domains of health care, disease prevention, and health promotion” [56], which were well-displayed in the reviewed instruments (Table 2). The association between health literacy and digital health use was not reported in the included studies. However, previous research suggests that health literacy is connected to the person’s tendency to search for health-related information on the internet [55,57] and the use of health apps and patient portals [58]. Based on these results, professionals could simultaneously promote the use of digital health by assessing clients’ health literacy and striving to improve a possible low literacy level. In addition to health literacy, this review suggests that attention should be paid to clients’ overall health-maintaining activity, including the skills and techniques to manage health issues and the ability to monitor their own condition and take appropriate steps for self-care. Moreover, the client’s familiarity with the health care system, concepts, and terminology was present in the instruments, which indicates that the ability to navigate the health care system is recognized as an important competency for clients to take advantage of digital health.

The role of dimensions related to attitudes and experiences was notably small in the instruments, given that previous studies have emphasized their importance in experiencing the benefits of digital health [12,59]. While 2 instruments included dimensions measuring clients’ motivation, confidence, and comfort to use digital health [36,38], a few more considered the perceived safety and trust issues of digital health [36,37,41,44]. Previous research especially supports the assessment of the latter aspects, as security, privacy, and confidentiality issues have been found to be considerable barriers to the use of digital health, especially among different vulnerable groups [12,60,61]. Another important finding in the examined instruments is their almost negligible focus on practical matters, such as the necessary equipment and facilities to use digital health, which were only considered in 1 instrument [42]. It is essential to ensure that the client can use digital health in a private setting, as it could prevent violations of personal data privacy and improve a secure user experience [12,62].

From the point of view of the practical usefulness of the instruments, the studies hardly provided any thresholds ​​for the values obtained (eg, what is considered high and low value) that could help professionals make informed decisions for clients’ care plans. The aspects of practical usefulness described in the studies were mainly based on the views of the authors. The most frequently mentioned aspects were the instrument’s ability to help professionals target the right digital solutions to the clients for whom they were suitable [27,30,31,38,41,42,44] and identify the clients who needed the most support, training, and assistance [29,36,39,40,44]. Only 1 study disclosed that, with the information obtained, professionals could better empower clients to take care of their own health with digital resources [26]. Based on the fact that the concept of empowerment has been increasingly discussed in connection with the rapid spread of digital health [63], it could have been expected to emerge more often in the studies. From a practical usefulness perspective, it is also important to consider that the concept of digital health encompasses a wide range of solutions and services, where client suitability, required skills, and other prerequisites can vary. For example, interactive services with professionals may demand different conditions than those used entirely independently and without interactive elements. Therefore, it is important to further consider the importance and applicability of the identified assessment areas when guiding clients to various digital services in the future.

Finally, it is worth considering whether the correct approach is to discuss the client’s suitability for using digital health and social care or whether the discussion should focus on the suitability of digital services for the clients. Developing digital services that are easy to use and accessible for everyone is a key goal [64], and equal access is a central (although only 1 among many) contributing factor in realizing digital health equity [65,66]. Digital health technologies have already increased the unequal distribution of health care services to some extent [67]. Thus, it is important that people are still offered the opportunity for face-to-face transactions if they do not have the necessary skills, willingness, or facilities to act digitally. The professionals’ role in making this assessment is crucial, and they need easy-to-use and valid tools that comprehensively assess the clients’ prerequisites for using digital solutions. It is still, however, important to note that the purpose of the assessment is not to exclude anyone from digital services but to help identify possible needs for support and take appropriate measures to remove barriers to use. Whether professionals have the required skills and sufficient time allocated for this in addition to their already heavy workload is another matter that requires further consideration.

### Limitations

Although we used several databases in the review, used the expertise of a research librarian, and worked in a group to discover eligible studies, it cannot be ruled out that some relevant instruments may have been missing. The lack of a manual search of relevant journals may have also limited the results of this review. Furthermore, this review excluded studies published in non-English journals, which may narrow the perspective on the topic. Finally, when using the conclusions of this review, one must acknowledge the limitations associated with the scoping review method, meaning the lack of formal quality appraisal of the included studies.

### Conclusions

This scoping review highlighted the need for comprehensive evaluation tools that can assess clients’ suitability for digital health and social care. While 12 assessment instruments were identified, none of them were developed specifically for social care. The dimensions included in the instruments emphasized the variety of required digital skills, a person’s general ability to maintain health, attitudes, and experiences, as well as practical matters such as equipment requirements. The studies also raised various aspects of the instruments’ practical usefulness, such as the ability to target appropriate digital solutions, identify support needs, and promote optimal use. However, the lack of defined suitability thresholds for values obtained (indicating a person’s likelihood of successfully using digital services) hinders informed decision-making when selecting suitable services for the client. Professionals play a crucial role in promoting clients’ use of digital technologies and need comprehensive and user-friendly tools to evaluate their ability to use digital solutions. In the future, it is necessary to further examine how different instruments and their dimensions predict clients’ use of digital health and social care. This information could ensure that the assessment would focus on the most relevant and essential factors related to the clients. Finally, the usability of the found instruments should be considered from the perspective of digital social services, as many themes (such as technical skills, remote communication, social support, existing facilities, or security issues) can equally be pivotal in supporting a client’s ability to benefit from digital social care. However, it is possible that the client profile in social services differs from those in need of health care, and digital social work possesses some unique characteristics that may not all be adequately captured by digital health dimensions. This emphasizes the possible need to develop and use separate assessment methods.

## Appendix

Multimedia Appendix 1 Search strategies and included studies.

Multimedia Appendix 2 PRISMA-ScR (Preferred Reporting Items for Systematic reviews and Meta-Analyses extension for Scoping Reviews) checklist.

## Abbreviations

CeHLS-D: Condition-Specific eHealth Literacy Scale for Diabetes
DHLI: Digital Health Literacy Instrument
DHLS: Digital Health Care Literacy Scale
DHTL-AQ: Digital Health Technology Literacy Assessment Questionnaire
eHEALS: eHealth Literacy Scale
eHL: eHealth literacy
eHLA: eHealth Literacy Assessment Toolkit
READHY: Readiness and Enablement Index for Health Technology
TeHLI: Transactional eHealth Literacy Instrument
