# Supplementary material for: Professionals Evaluating Clients’ Suitability for Digital Health and Social Care: Scoping Review of Assessment Instruments
Source: J Med Internet Res. 2023 Nov 30;25:e51450. doi: 10.2196/51450 (PMC10722370; doi:10.2196/51450)
Supplement: Multimedia Appendix 1 [file jmir_v25i1e51450_app1.docx]

Multimedia Appendix 1.

**Table S1.** Search strategy for databases.

| **Databases** | **Search terms** | **Hits** |
| --- | --- | --- |
| **Web of Science** |  |  |
| 1 | AB=((e-health OR ehealth OR "digital health" OR "e-service" OR "e-services" OR "digital service" OR "digital services" OR "digital social" OR e-social OR esocial OR "electronic social" OR telemedicine OR telehealth) NEAR/3 (literacy OR suitability OR eligibility OR appropriat* OR confidence OR confident OR capab* OR skills OR able OR abilit* OR "self efficacy" OR readiness OR acceptance OR adoption)) | 2437 |
| 2 | TI=((e-health OR ehealth OR "digital health" OR "e-service" OR "e-services" OR "digital service" OR "digital services" OR "digital social" OR e-social OR esocial OR "electronic social" OR telemedicine OR telehealth) NEAR/3 (literacy OR suitability OR eligibility OR appropriat* OR confidence OR confident OR capab* OR skills OR able OR abilit* OR "self efficacy" OR readiness OR acceptance OR adoption)) | 876 |
| 3 | #1 OR #2 | 2748 |
| 4 | AB=(((literacy OR suitab* OR eligib* OR appropriat* OR confidence OR confident OR capab* OR skills OR able OR abilit* OR "self efficacy" OR readiness OR acceptance OR adoption) NEAR/3 (evaluat* OR measur* OR assess* OR psychometric* OR scale* OR questionnaire* OR test* OR screening OR tool))) OR TI=(((literacy OR suitab* OR eligib* OR appropriat* OR confidence OR confident OR capab* OR skills OR able OR abilit* OR "self efficacy" OR readiness OR acceptance OR adoption) NEAR/3 (evaluat* OR measur* OR assess* OR psychometric* OR scale* OR questionnaire* OR test* OR screening OR tool))) | 345025 |
| 5 | AB=(student* or teach*) or TI=(student* or teach*) | 699019 |
| 6 | #3 AND #4 NOT #5 | 513 |
| **ASSIA** |  |  |
| 1 | abstract(((e-health OR ehealth OR "digital health" OR "e-service" OR "e-services" OR "digital service" OR "digital services" OR "digital social" OR e-social OR esocial OR "electronic social" OR telemedicine OR telehealth) NEAR/3 (literacy OR suitability OR eligibility OR appropriat* OR confidence OR confident OR capab* OR skills OR able OR abilit* OR "self efficacy" OR readiness OR acceptance OR adoption)) .) OR title(((e-health OR ehealth OR "digital health" OR "e-service" OR "e-services" OR "digital service" OR "digital services" OR "digital social" OR e-social OR esocial OR "electronic social" OR telemedicine OR telehealth) NEAR/3 (literacy OR suitability OR eligibility OR appropriat* OR confidence OR confident OR capab* OR skills OR able OR abilit* OR "self efficacy" OR readiness OR acceptance OR adoption)) .) AND pd(2012-2023) | 328 |
| 2 | abstract(((literacy OR suitab* OR eligib* OR appropriat* OR confidence OR confident OR capab* OR skills OR able OR abilit* OR "self efficacy" OR readiness OR acceptance OR adoption) NEAR/3 (evaluat* OR measur* OR assess* OR psychometric* OR scale* OR questionnaire* OR test* OR screening OR tool))) OR title(((literacy OR suitab* OR eligib* OR appropriat* OR confidence OR confident OR capab* OR skills OR able OR abilit* OR "self efficacy" OR readiness OR acceptance OR adoption) NEAR/3 (evaluat* OR measur* OR assess* OR psychometric* OR scale* OR questionnaire* OR test* OR screening OR tool))) AND pd(2012-2023) | 13593 |
| 3 | 1 AND 2 | 112 |
| 4 | abstract(student* or teach*) OR title(student* or teach*) | 140297 |
| 5 | 3 NOT 4 | 91 |
| **Medline (OVID)** |  |  |
| 1 | ((e-health or ehealth or "digital health" or "e-service" or "e-services" or "digital service" or "digital services" or "digital social" or e-social or esocial or "electronic social" or telemedicine or telehealth) adj3 (literacy or suitability or eligibility or appropriat* or confidence or confident or capab* or skills or able or abilit* or "self efficacy" or readiness or acceptance or adoption)).ti,ab,kf. | 2607 |
| 2 | ((literacy or suitab* or eligib* or appropriat* or confidence or confident or capab* or skills or able or abilit* or "self efficacy" or readiness or acceptance or adoption) adj3 (evaluat* or measur* or assess* or psychometric* or scale* or questionnaire* or test* or screening or tool)).ti,ab,kf. | 290901 |
| 3 | limit 2 to yr="2012 -Current" | 169202 |
| 4 | 1 and 3 | 509 |
| 5 | Telemedicine/ | 35958 |
| 6 | Health Literacy/ or Self Efficacy/ | 32430 |
| 7 | Psychometrics/ | 87126 |
| 8 | 5 and 6 and 7 | 33 |
| 9 | 4 or 8 | 512 |
| 10 | student*.mp. [mp=title, book title, abstract, original title, name of substance word, subject heading word, floating sub-heading word, keyword heading word, organism supplementary concept word, protocol supplementary concept word, rare disease supplementary concept word, unique identifier, synonyms] | 387626 |
| 11 | 9 not 10 | 418 |
| **CINAHL** |  |  |
| 1 | TI ( (e-health or ehealth or "digital health" or "e-service" or "e-services" or "digital service" or "digital services" or "digital social" or e-social or esocial or "electronic social" or telemedicine or telehealth) N3 (literacy or suitability or eligibility or appropriat* or confidence or confident or capab* or skills or able or ability* or “self efficacy” or readiness or acceptance or adoption) ) OR AB ( (e-health or ehealth or "digital health" or "e-service" or "e-services" or "digital service" or "digital services" or "digital social" or e-social or esocial or "electronic social" or telemedicine or telehealth) N3 (literacy or suitability or eligibility or appropriat* or confidence or confident or capab* or skills or readiness or acceptance or adoption) ) | 1565 |
| 2 | TI ( (literacy or suitab* or eligib* or appropriat* or confidence or confident or capab* or skills or able or abilit* or "self efficacy" or access* or readiness or acceptance or adoption) N3 (evaluat* or measur* or assess* or psychometric* or scale* or questionnaire* or test*) ) OR AB ( (literacy or suitab* or eligib* or appropriat* or confidence or confident or capab* or skills or able or abilit* or "self efficacy" or access* or readiness or acceptance or adoption) N3 (evaluat* or measur* or assess* or psychometric* or scale* or questionnaire* or test* or tool) ) | 79477 |
| 3 | (MH "Telemedicine") OR (MH "Telerehabilitation") OR (MH "Telepsychiatry") OR (MH "Telehealth") OR (MH "Remote Consultation") OR (MH "Telenursing") OR (MH “Digital Health”) OR (MH “Digital Technology”) | 36350 |
| 4 | (MH "Health Literacy") OR (MH “Information Literacy”) | 11415 |
| 5 | (MH "Measurement Issues and Assessments") OR (MH "Psychometrics") OR (MH “Instrument Construction) OR (MH “Instrument Validation”) | 35686 |
| 6 | student* | 275606 |
| 7 | 1 AND 2 | 412 |
| 8 | 3 AND 4 AND 5 | 23 |
| 9 | (7 OR S) NOT 6 | 324 |

**Table S2.** Studies included in the review.

| **Author(s), year, country** | **Aim** | **Methods and participants** | **Measure(s), dimensions, number of items** | **Associations with eHealth use / other findings** | **Practical usefulness for professionals** |
| --- | --- | --- | --- | --- | --- |
| Chang & Schulz 2018 [26]  China | To compare the Chinese eHEALS (C-eHEALS) with other eHEALS findings to determine whether the C-eHEALS model provided reliable evidence with chronic disease patients. | Online survey,  patients in a hospital outpatient department (n=352) | Chinese electronic Health Literacy Scale  (C-eHEALS),  Acquiring information from the Internet, 1 factor, 8 items | Cluster analysis separated High and Low eHealth literacy groups. The high eHealth literacy group had significantly higher computer and technology skills and was more interested in using media and other online information channels than the low eHealth literacy group which favoured face-to-face inquiries for getting information. No cut-off values were given for high/low eHealth literacy groups. | C-eHEALS was stated to be a valid and reliable measure of eHealth literacy among patients with chronic disease in China, and healthcare providers can use it as a screening tool and empower patients to take care of their health using online resources. |
| Chen et al., 2022 [34]  Taiwan | To adapt the eHealth Literacy Questionnaire (eHLQ) for application among people with chronic diseases and conduct validity-testing procedures / explore its psychometric properties. | Questionnaire study, patients with one or more chronic diseases in different outpatient department units at several hospitals (n=440) | eHealth Literacy Questionnaire (eHLQ)  1) using technology to process health information, 5 items  2) understanding of health concepts and language, 5 items  3) ability to actively engage with digital services, 5 items 4) feel safe and in control, 5 items  5) motivated to engage with digital services, 5 items  6) access to digital services that work, 6 items  7) digital services that suit individual needs, 4 items | Associations between eHLQ and eHealth use were not examined.  The eHLQ showed good psychometric properties, and the 7 scales were stated to be useful in assessing different dimensions of eHealth literacy among people with chronic diseases. | There was no mention of the usefulness of the eHLQ in professionals’ work. However, the questionnaire may enable eHealth system developers and healthcare providers to understand people’s ability to engage with and use technology, which can help in developing, evaluating and redesigning the systems. The eHLQ can also be used to improve the quality/effectiveness of care by designing more adaptive care programmes. |
| Cheng et al., 2022 [35]  Australia | To evaluate the content, response process and internal structure / psychometric properties of the eHealth Literacy Questionnaire (eHLQ) | Cognitive interviews (n=12),  Cross-sectional survey, community health setting, clients from two private primary care medical clinics (n=525) | eHealth Literacy Questionnaire (eHLQ)  1) using technology to process health information, 5 items  2) understanding of health concepts and language, 5 items  3) ability to actively engage with digital services, 5 items 4) feel safe and in control, 5 items  5) motivated to engage with digital services, 5 items  6) access to digital services that work, 6 items  7) digital services that suit individual needs, 4 items | Associations between eHLQ and eHealth use were not examined.  The items were generally well-understood and the eHLQ demonstrated robust psychometric properties (although some potential weaknesses in discriminant validity were shown). | The eHLQ was stated to be a useful tool for different parties ( healthcare providers, researchers, digital health developers, policymakers) and help in understanding the eHealth literacy needs of patients and in developing, implementing and evaluating digital health interventions that better match users’ health needs. |
| Dale et al., 2020 [28]  Norway | To examine the measurement properties of the Norwegian version of the Electronic Health Literacy Scale (eHEALS) among patients undergoing day surgery. | Cross-sectional survey, patients undergoing day surgical treatment (n=109) | Electronic Health Literacy Scale (eHEALS)  8 items 🡪 2 factors (based on CFA):  1) Information awareness, 5 items  2) information engagement, 3 items | Associations between eHEALS and eHealth use were not examined.  The measurement properties of the Norwegian version of the eHEALS showed good internal consistency, and it can be an appropriate tool for measuring eHealth literacy in day surgery patients. | eHEALS is concise and easy for professionals to use. Due to the short treatment time, it is important for professionals to be aware of the patients' eHealth literacy level so that they can ensure that the patient has the skills and opportunities to use digital services in self-care after the procedure. |
| He et al., 2021 [37]  China | To test the reliability and validity of the translated eHealth Literacy Scale (e-HLS) among stroke patients in China. | Cross-sectional survey of stroke patients from one hospital (N=648) | Chinese eHealth Literacy Scale (e-HLS-CHI), 19 items  🡪 3 dimensions (based on CFA):  1) Action, 13 items  2) Trust, 4 items  3) Communication, 2 items.  eHEALS was used to test concurrent validity. | The e-HLS showed good reliability and validity. When testing the predictive validity of e-HLS, scores from the eHEALS (max. score 40) were divided into higher (total score ≥ 20) and lower level eHealth literacy (total score < 20). Based on the ROC curve, the cut-off point in e-HLS was 32 points (score separating low/high eHealth literacy) | Findings about the scale may be useful for clinical nurses in assessing the eHealth literacy of stroke patients. This knowledge is needed to understand how the patients identify, judge and use online health resources. And it can help in designing future health promotion programs. |
| Hyde et al., 2018 [29] Australia | To test the factorial validity and internal consistency of the three-factor eHEALS structure among magnetic resonance imaging (MRI) and computed tomography (CT) medical imaging outpatients. | Cross-sectional survey, MRI and CT imaging outpatients from one hospital (n=256) | eHEALS, 8 items🡪 3 factors:  1) Awareness, 2 items  2) Skills, 3 items  3) Evaluate, 3 items.    + | Data supported the three-factor structure of eHEALS.  However, the fit of the model improved when item 3, ‘I know how to find helpful health resources on the Internet’, was excluded🡪 an adapted 7-item eHEALS could be considered after further testing. | Healthcare professionals can use the eHEALS to identify low competency areas (awareness, skills and evaluation) and target eHealth literacy improvement interventions. For example, professionals can recommend Web-based sources to increase patients’ awareness and reduce their need to evaluate contents or arrange training sessions and give advice and provide checklists on how to evaluate Web-based sources.  Based on the results, appropriate support and assistance can be directed towards those individuals who require it the most.  The eHEALS may not be suitable for those patients who do not use the Internet for health. |
| Karnoe et al., 2018 [38]  Norway | To develop and validate an eHealth literacy assessment toolkit (eHLA) | Questionnaire study. Patients from an outpatient clinic at one hospital (n=100) and a sample from the general population (n=375) | eHealth literacy assessment toolkit (eHLA)combines 7 instruments, of which 4 evaluate competencies related to health, and 3 evaluate digitally related competencies:  1) familiarity with technology, 6 items (final version after testing)  2) technology confidence, 4 items (final version)  3) incentives for engaging with technology (i.e., motivation), 4 items (final version) | The eHLA tools showed satisfactory validity and objectivity.  Associations between the tools and use of eHealth were not assessed, but the need was mentioned: | The eHLA toolkit can be used to screen and assess the eligibility of an individual’s participation in projects involving eHealth solutions.  The three digitally related tools were highly correlated. Thus, all three tools may not be needed, but one of the three could be chosen depending on whether there is a greater need for assessing an individual’s familiarity with technology, technology confidence or motivation. |
| Kayser et al., 2019 [36]  Denmark | To evaluate if the combination of eHealth Literacy Questionnaire (eHLQ), selected dimensions from the Health Education Impact Questionnaire (heiQ) and the Health Literacy Questionnaire (HLQ) can be used as a joint instrument to characterize an individual’s level of health technology readiness, and whether different health technology readiness profiles can be created from the data of potential users of health technologies and digital health services. | Cross-sectional study (paper form survey), patients with a recent cancer diagnosis referred to rehabilitation (n=305) | Readiness and Enablement Index for Health Technology (READHY) instrument, 13 dimensions and 65 items from:  1) eHealth Literacy Questionnaire (eHLQ)  2) Health Education Impact Questionnaire (heiQ) (selected dimensions)  3) Health Literacy Questionnaire (HLQ) | Discriminant validity between some of the dimensions/scales was not sufficient. Reducing the number of items did not lead to a better fit.  More evidence is needed about which scales predict future health technology readiness and other targeted outcomes in different settings. | The READHY instrument can be used to evaluate the readiness and ability of an to engage with and take advantage of technologies.  Reducing dimensions would be useful (long survey), or professionals can choose only specific dimensions with proper justification or hypotheses.  Identified readiness profiles may be useful to identify those at risk of being marginalised or needing specific interventions. |
| Lee et al., 2022 [39]  South Korea | Test the psychometric properties of a developed Condition-Specific eHealth Literacy Scale for Diabetes (CeHLS-D). | Patients with diabetes (n=453) from outpatient clinics at multiple hospitals. | Condition-Specific eHealth Literacy Scale for Diabetes (CeHLS-D), 10 items 🡪 2 dimensions (based on EFA/CFA):  1) Cognitive actions for Internet diabetes information, 7 items  2) Abilities of digital communication, 3 items | The instrument was stated to be closer to a generic than to a condition-specific instrument.  Associations between the scale and the use of eHealth were not assessed. | The CeHLS-D can be used in practice for diabetic patients regardless of gender, age and glycemic control status. Due to the low number of items, the CeHLS-D may also be feasible for use in practice, and it is not too burdensome to respondents.  Professionals can use the instrument to identify patients with low eHealth literacy and better inform these patients about how to avoid obtaining conflicting or misleading diabetes information from the Internet and guide them to trustworthy sources. |
| Lin et al., 2020 [30]  Iran | To investigate the psychometric properties of the Persian version of the eHealth Literacy Scale (eHEALS) among people with heart failure. | Patients diagnosed with heart failure (n=388) from three university hospitals. | eHealth Literacy Scale (eHEALS), 8 items🡪 unidimensional structure (based on Rasch analysis). | The results of the study demonstrated promising psychometric properties of the eHEALS among patients with heart failure. | Healthcare providers can use the eHEALS score to make clinical decisions on whether patients with heart failure should use and have the ability to use online resources (safely) for health improvement and maintenance. Or should face-to-face intervention/guidance, such as personal patient education about self-care, be provided to them? |
| Nelson et al., 2022 [40]  USA | To assess the validity of the developed 3-item Digital Healthcare Literacy Scale (DHLS). | Survey study. Caregivers of young children visiting a pediatric primary care clinic at a university medical centre (n=508). | Digital Healthcare Literacy Scale (DHLS), 3 items:  Measures the basic skills necessary for using digital health services (mainly telehealth but can also be suitable for other services, such as patient portals). | The DHLS showed strong psychometric properties. A lower healthcare literacy score was associated with less experience with digital healthcare and a lower likelihood of owning digital tools. | The DHLS can be used in clinical settings as a brief assessment of individuals’ ability to use telehealth. A high score may indicate that the person is a strong candidate for telehealth. Those with a lower score can be provided additional assistance to be better equipped for a visit.  DHLS is a screening tool that helps to identify those most in need of support and training to use telehealth so that (expensive) resources are not ‘wasted’, thus, promoting efficiency and effectiveness.  The scale can also help to identify patients who may be unaware that they need assistance or feel uncomfortable communicating their need for help. |
| Noblin et al., 2012 [31]  USA | To examine whether demographic factors and measuring patients’ e-health literacy (with eHEALS) could help in determining whether patients are willing to use (intending to adopt) a personal health record (PHR). | Cross-sectional survey, patients visiting family practice physicians and internal medicine physicians in a six-week period (n=562) | eHealth Literacy Scale (eHEALS), 8 items🡪 unidimensional structure | Patients with a high level of eHealth literacy were more likely to adopt and use a PHR compared to patients with a lower level of eHealth literacy.  Statements that correlated the most with an intention to adopt the PHR were: ‘I know how to use the Internet to answer my questions about health’ and ‘I know how to use the health information I find on the Internet to help me’. | When planning to provide a PHR for patients, physicians should look beyond patients’ demographic characteristics.  eHEALS can help physicians to know how likely their patients are to use PHR in the future. |
| Paige et al., 2017 [27]  USA | To explore the one-factor structure and reliability of eHEALS scores among patients with chronic disease who report using the Internet to find health information. | Web-based survey, patients with one or more chronic diseases reporting that they have used the Internet during the past 12 months (n=649) | eHealth Literacy Scale (eHEALS), 8 items🡪 unidimensional structure | eHEALS was shown to be reliable among the study population, and the study supported its one-factor structure.  Patients with higher eHealth literacy were more likely to ‘agree/strongly agree’ that they have the self-efficacy and knowledge to locate, understand and act on health information from electronic sources/the Internet  It was recommended that updating the items could be useful so that they would better consider the dynamic and interactive (participatory nature of) online health information.  Future studies should explore convergent and divergent validity evidence by examining associations between eHEALS scores and frequency of Internet use (among other outcomes, such as a patient’s engagement in health-promoting behaviours). | eHEALS is a brief scale that can be used as (part of) a screening and diagnostic instrument that can make healthcare providers (1) more aware of their patients’ eHealth literacy skills to recommend or prescribe the online resource that will promote their self-care and well-being and (2) identify patients who need training to enhance their eHealth literacy skills. |
| Paige et al., 2019 [41]  USA | To test the psychometric properties of the developed multi-dimensional Transactional eHealth Literacy Instrument (TeHLI). | Web-based survey, Patients with a COPD diagnosis enrolled in a large university research registry (n=283). | Transactional eHealth Literacy Instrument (TeHLI), 18 items🡪 4 dimensions:  1) Functional eHealth literacy (= basic skills in reading and typing about health effectively on the Internet), 4 items  2) Communicative eHealth literacy (= the ability to collaborate, adapt and control communication about health with users in social online environments with multimedia), 5 items  3) Critical eHealth literacy (=the ability to evaluate the credibility, relevance and risks of sharing and receiving health information on the Internet), 5 items  4) Translational eHealth literacy (=the ability to apply health knowledge gained from the Internet across diverse ecological contexts), 4 items. | Of the TeHLI scales, communicative, critical and translational skills were associated with the number of social media used for health.  On each scale, a higher score was associated with higher active and interactive online health information-seeking, higher perceived usefulness of the Internet for health-related purposes and lower online information-seeking challenges.  The strongest correlation was between communicative skills and interactive online health information-seeking behaviours. | The TeHLI allows healthcare practitioners to assess the four dimensions from their patients to see which are the potential strengths and deficits (for example, if someone only has the skills to review online websites but may not be able to engage in exchanges among other users). The TeHLI can be useful for practitioners to direct their patients to services/resources that match their eHealth attitudes, preferences and skills. |
| Richard et al., 2023 [42]  Canada | To create a screening tool for music therapists to evaluate an autistic person’s suitability for telehealth. | Survey with open-ended questions, music therapists (n=192) | Telehealth music therapy screening tool for autistic individuals, 19 individual items  Part A, Client characteristics,  e.g., does the individual have limited access to in-person music therapy? Is the individual interested in telehealth? Does the client have reliable Internet connections and access to a computer with a Web camera & microphone? Is the individual familiar with screens/computer usage? Is the individual’s environment calm and free from distractions? Does the individual have an appropriate space for sessions (e.g. space for movement when addressing sensorimotor goals)? Does this individual tend to suffer from social anxiety when in the physical presence of others?  Part B, Therapist characteristics. | The factors that may determine whether autistic persons are likely to be suitable for music therapy over telehealth are summarised as  the client’s ‘*comfort level with screens, the level of distraction in the home, the presence and effectiveness of caregivers, the ability to build rapport with the client, the client’s age, level of verbal ability, sensory needs, presence of technology resources, prior social anxiety and the overall ability to attend telehealth sessions’*. | By assessing the identified factors, music therapists can identify which clients are more (or less) suitable for telehealth. Using the screening tool can help therapists make telehealth music therapy more effective for autistic persons and maximize their ability to provide music therapy for clients over telehealth. |
| Richtering et al., 2017 [32]  Australia | To examine the internal construct validity of an eHealth and health literacy scale (eHEALS and HLQ) in a population with moderate to high cardiovascular disease risk | Survey study, patients with moderate to high cardiovascular disease risk (n=397) | eHealth Literacy Scale (eHEALS), 8 items🡪 2 dimensions (based on principal components analysis),  1) Knowledge about resources, 5 items  2) Evaluation of resources, 3 items  Health literacy questionnaire (HLQ), 9 dimensions (9 separate scores)  1) Feeling understood and supported by the healthcare provider, 4 items  2) Having sufficient information to manage my health, 4 items  3) Actively managing my health, 5 items  4) Social support for health, 5 items  5) Appraisal of health information, 5 items  6) Ability to actively engage with healthcare providers, 5 items  7) Navigating the healthcare system, 6 items  8) Ability to find good health information, 5 items  9) Understanding health information well enough to know what to do, 5 items | eHEALS demonstrated good psychometric properties and results supported its use as a measure of eHealth literacy, with a higher score indicating higher levels of eHealth literacy. HLQ was reported to measure its nine dimensions appropriately.  Both measurements’ ability to measure change over time was found problematic because respondents would have to acquire a considerable increase in eHealth literacy and HLQ to be reflected in their scores.  (HLQ may require re-scoring of the response categories in dimensions 6–9.)  Two dimensions of eHealth literacy may need to be assessed/scored separately.  Further studies are needed to determine the precision of eHEALS in identifying people with greater or lower eHealth capacities. | Healthcare providers need valid and reliable scales to assess a patient’s eHealth literacy and health literacy. |
| Seon- Yoon & Eun-Shim, 2015 [33]  USA | To test the psychometric aspects of the eHEALS for older adults. | Secondary data analysis (two measuring points), older adults participating in an online bone health intervention study (n=866) | eHealth Literacy Scale (eHEALS), 8 items, unidimensional structure (based on exploratory factor analysis) | eHEALS was shown to be reliable and valid among older adults. Further studies should be conducted with samples with other disease conditions.  eHealth literacy significantly correlated with the use of the Internet. | Healthcare providers can use the eHEALS as a convenient screening tool to assess the eHealth literacy of older adults and optimize the benefits of eHealth programs with interventions that facilitate patients’ access, understanding and use of information as best as they can. |
| Yoon et al., 2022 [43]  Korea | Psychometric validation of a developed Digital Health Technology Literacy Assessment Questionnaire (DHTL-AQ). | Survey study, patients with (n=377) and without a chronic disease (n=213) | Digital Health Technology Literacy Assessment Questionnaire (DHTL-AQ), 34 items🡪 2 domains: digital functional literacy and digital critical literacy  4 categories:  1) ability to use an app  2) knowledge of app icons  3) ICT-related terms  4) evaluating the reliability and relevance of digital health (health information)  Short version DHTL-AQ, 20 items🡪 1 domain (digital functional literacy), 2 categories:  1)ICT terms  2) use of an app | The DHTL-AQ correlated strongly (0.76) with actual performance skills / task ability when using a mobile app. To compare, the correlation between eHealth literacy and task ability was 0.41. | DHTL-AQ was developed to be used in clinical settings, and especially the short version can be a helpful screening tool to evaluate patients’ DHTL in a busy clinical setting.  It can be easily completed by vulnerable digital health groups because the questionnaire is simple and includes terms and icons. |
| Van der Vaart & Drossaert 2017 [44]  The Netherlands | To examine the reliability and validity of a developed Digital Health Literacy Instrument (DHLI) that includes both self-reported and performance-based items. | Paper and pen survey, general Dutch population (n=200) | Digital Health Literacy Instrument (DHLI),  21 self-reported items🡪 7 dimensions:  1) operational skills to use the computer and Internet browser  2) information-searching skills to use correct search strategies  3) evaluating the reliability of online information  4) determining the relevance of online information  5) navigation skills to navigate and orientate on the Web  6) adding self-generated content to Web-based apps  7) protecting and respecting privacy while using the Internet.  + 7 performance-based items (one per dimension) testing the participant’s ability to apply a particular skill in a fictional situation. | The reliability and validity of the self-report scale of the DHLI were sufficient.  The performance-based items propose a new way to measure actual digital health literacy skills, but their applicability should be developed further to see their value.  DHLI significantly correlated with Internet use (0.39 p <0.001) and health-related Internet use (0.27 p<0.001). | In daily clinical practice, for example, the tool could help make decisions about how much a patient can benefit from specific digital health tools and treatments. Additionally, it could help guide and train patients who require assistance in using online health tools. |
